# A systematic literature review of economic evaluations and cost-of-illness studies of inherited cardiomyopathies

**DOI:** 10.1007/s12471-023-01776-1

**Published:** 2023-05-12

**Authors:** Isabell Wiethoff, Birgit Goversen, Michelle Michels, Jolanda van der Velden, Mickaël Hiligsmann, Tom Kugener, Silvia M. A. A. Evers

**Affiliations:** 1 Department of Health Services Research, Care and Public Health Research Institute (CAPHRI), Maastricht University, Maastricht, The Netherlands; 2 Department of Physiology, Amsterdam UMC, Vrije Universiteit, Amsterdam Cardiovascular Sciences Institute, Amsterdam, The Netherlands; 3 Department of Cardiology, Thoraxcenter, Erasmus MC Rotterdam, Rotterdam, The Netherlands; 4 Centre for Economic Evaluation and Machine Learning, Trimbos Institute, Netherlands Institute of Mental Health and Addiction, Utrecht, The Netherlands

**Keywords:** Cost of illness, Economic evaluation, Dilated cardiomyopathy, Hypertrophic cardiomyopathy, Systematic review

## Abstract

Hypertrophic cardiomyopathy (HCM) and dilated cardiomyopathy (DCM) are commonly inherited heart conditions associated with a high risk of heart failure and sudden cardiac death. To understand the economic and societal disease burden, this study systematically identified and reviewed cost-of-illness (COI) studies and economic evaluations (EEs) of various interventions for HCM and DCM. A literature search was performed in MEDLINE, EMBASE, NHS EED, EconLit and Web of Science to identify COI studies and EEs published between 1 January 2010 and 28 April 2021. The selection of studies and their critical appraisal were performed jointly by two independent researchers. For the quality assessment, the ‘Consensus on Health Economic Criteria’ list was used. Two COI studies and 11 EEs were eligible for inclusion. Cost-effectiveness varied among interventions and depended on the targeted patient population. Both COI studies identified only hospitalisation costs in HCM. The mean study quality was high in EEs but low in COI studies. Most studies excluded costs for patients, caregivers and productivity losses. Overall, knowledge of the societal and economic burden of inherited cardiomyopathies is limited. Future research needs to include quality-adjusted life years and a broader range of costs to provide an information base for optimising care for affected patients.

**Supplementary Information:**

The online version of this article (10.1007/s12471-023-01776-1) contains supplementary material, which is available to authorized users.

## Introduction

Cardiomyopathies (CMs) are a group of structural and functional disorders of the heart muscle associated with a high risk of heart failure (HF) and sudden cardiac death (SCD) [1]. The most frequent forms are dilated cardiomyopathy (DCM) and hypertrophic cardiomyopathy (HCM), affecting roughly 0.4% and 0.2% of the general population, respectively [2]. CMs belong to the most common inheritable heart conditions, and a pathogenic DNA variant is identified in ~ 20% of DCM and 30–60% of HCM cases [2–4]. As most CMs are inherited in an autosomal dominant manner, first-degree family members have a ~ 50% chance of carrying the genetic defect, with a large heterogeneity in phenotypic expression [1]. Due to the often asymptomatic clinical course of the disease’s early stages, it tends to remain unnoticed [1]. Family screening for CM focuses on the early detection of family members at risk, by using both genetic and cardiac screening methods to identify the disease early and to enhance the prevention of SCD and disease progression. In individuals with a severe phenotype, invasive and thus expensive treatments such as myectomy, alcohol septal ablation, implantable cardioverter defibrillators (ICDs) and heart transplantations are needed to reduce the burden of disease [1, 2, 4]. In addition, the psychosocial strain due to anxieties and intensive lifestyle changes plays a major role for patients and family members, leading to losses in quality of life [1, 5]. Therefore, inherited CMs can have a substantial impact on the morbidity and mortality of patients of all age groups, leading to an economic and societal burden.

To optimise care for patients and relatives in whom a genetic defect has been identified, it is essential to understand the economic impact and the cost drivers of HCM and DCM. Cost-of-illness (COI) studies are important tools for quantifying the economic burden by estimating the use of healthcare resources, costs and productivity losses engendered by the inability to work [6]. In addition, economic evaluations (EEs) are increasingly being conducted to assess the cost-effectiveness of various interventions [7]. In full EEs, at least two interventions are assessed according to their costs and benefits by calculating an incremental cost-effectiveness ratio (ICER) [7]. Costs are measured in monetary units, while effects are usually measured in quality-adjusted life years (QALYs) [7]. Thereby, it is possible to determine the most promising interventions for reducing the burden of disease for affected patients and for society [7]. Especially in high-income countries, EEs are a widely used instrument to inform policy-makers and to facilitate reimbursement decisions [7].

**Fig. 1:**
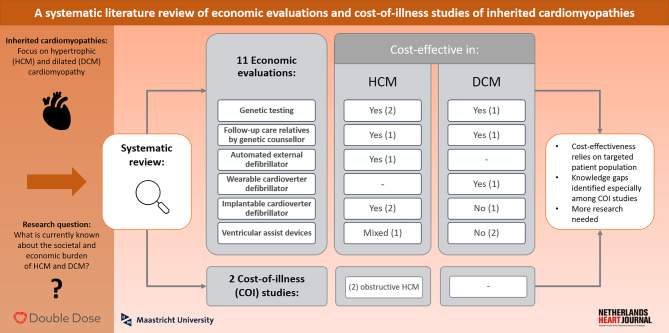
Infographic

While various EEs and COI studies exist for more general heart diseases, such as HF, the number of studies specifically addressing inherited CMs is more limited [8–11]. Even though few studies exist, an overview of the available literature is missing, as no previous study has yet systematically reviewed costs and cost-effectiveness in HCM and DCM [9, 11]. Such an overview, however, can be highly valuable, as it summarises the existing literature in a useful manner while revealing literature gaps, which may guide future research. Further, a summary of the currently available knowledge can function as a meaningful basis for decision-making [12]. Thus, the aim of this study is to provide an overview of health economics studies dealing with the societal and economic consequences of inherited CMs and to disclose potential knowledge gaps. Accordingly, a systematic literature review was performed to identify COI studies and full EEs of current interventions for HCM and DCM (Fig. 1).

## Methods

The present review is part of the Dutch Double Dose research project, which aims to investigate the influence of metabolic stress on the pathophysiology of inherited CMs, in order to optimise diagnosis and treatment for CM patients [13]. For methodological guidance, the five-step approach used by van Mastrigt and colleagues was followed [14–16]. Transparent reporting was ensured by complying with the Preferred Reporting Items for Systematic Reviews and Meta-Analyses (PRISMA 2020) guidelines [17]. A detailed study protocol was published in the International Prospective Register of Systematic Reviews (PROSPERO) under registration number CRD42021248484 [18].

### Eligibility criteria

Eligibility criteria were selected based on the PICO (Population—Intervention—Comparator—Outcome) mnemonic [14]. Studies dealing with (P) HCM or DCM patients or relatives at risk, (I & C) any diagnostic or treatment intervention for inherited CMs and (O) cost-effectiveness or cost-utility of different interventions or healthcare costs, costs for patients and caregivers or productivity losses were included in evidence synthesis. Studies published ≥ 2010 in English or German with a focus on high-income countries as defined by the World Bank were included [19]. Letters, expert opinions, editorials, conference abstracts and reviews were excluded. Relevant reviews were considered for reference checking.

### Data collection and screening

The literature search was performed in MEDLINE, EMBASE, National Health Service Economic Evaluation Database (NHS EED), EconLit (EBSCO) and ‘Web of Science’ (SCI). The NHS EED stopped its service in March 2015 and is no longer updated; however, the database might still contain relevant EEs [15]. The search strategy consisted of keyword components for the disease, EEs and COI studies. Based on previously conducted searches, the disease-related component was kept very broad to include non-specific interventions for inherited and non-inherited CMs. For the component ‘economic evaluations’, pre-validated search filters of the Canadian Agency for Drugs and Technologies in Health (CADTH) were employed and complemented with COI study-specific keywords by the Boolean operator ‘OR’ [20, 21]. A 10-year filter and a limiter for human studies was used to identify relevant up-to-date literature. The final search strategy was conducted on 28 April 2021 and can be found in Section A of the Electronic Supplementary Material. After removing duplicates, all records were scanned by title and abstract. Then full-text analyses were performed by at least two independent reviewers (double scoring). In case of doubt, a third reviewer was consulted. In eligible studies, reference checking was conducted to complement the database research. For literature management, EndNote X9.3.3. was used.

### Data extraction and quality assessment

Relevant study characteristics, including title, author, year, journal, country, perspective, study population, intervention, comparators, methods (model type, outcomes, time horizon, discount rates, reference year), results, sensitivity analyses and funding were summarised in a data extraction sheet. During data extraction, all cost data and ICERs were converted into 2020 Euros by using the free web-based tool of the Campbell and Cochrane Economics Methods Group (CCEMG) and the Evidence for Policy and Practice Information and Coordinating Centre (EPPI-Centre) [22]. If the reference year was missing, the publication date of the study was adopted. Results for EEs and COI studies were synthesised separately and presented by distinguishing between different interventions and patient subgroups. Results reported in EEs were summarised in a cost-effectiveness plane. Thereby, the cost-effectiveness of a certain intervention was visualised by showing cost differences on the vertical axis and effect differences on the horizontal axis [7]. To ease interpretation, a higher and a lower willingness to pay (WTP) threshold of € 100,000 and € 50,000 per QALY gained were included. Interventions that lie below these thresholds are considered cost-effective [7].

For the quality assessment of EEs, the extended Consensus on Health Economic Criteria (CHEC-extended) list was used. The methodological quality of each study was analysed by answering 20 yes-or-no questions [16, 23]. For COI studies, a modified version of the CHEC list with 13 yes-or-no questions was employed [6, 23]. A quality score was calculated for each study by assigning the value 1 to questions answered with yes and values 0.5 or 0 to questions which were suboptimal or not answered. All quality scores were double scored independently and discrepancies were resolved in consensus meetings. Both checklists are included in Section B of the Electronic Supplementary Material.

## Results

**Fig. 2:**
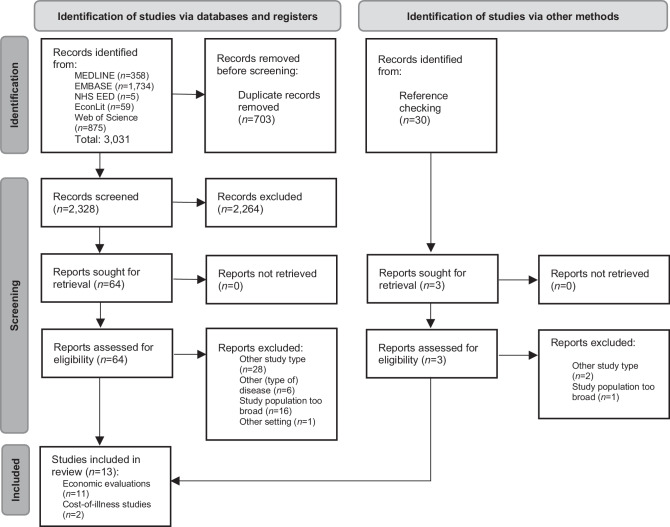
Extended PRISMA 2020 flowchart

Overall, 3031 studies were identified through the database search. After deduplication, 2328 studies were screened according to title and abstract. Sixty-four studies were assessed for eligibility via full-text reading. Reference checking was performed in 30 articles which were identified during the selection process. The full text of three additional sources was analysed, but considered irrelevant after assessment. Finally, 13 studies were identified as eligible and considered for evidence synthesis, 11 EEs and two COI studies. The most frequent reasons for exclusion were a different study type (*n* = 28), a too broad study population (*n* = 16), a different disease (*n* = 6), or a policy analysis model, here referred to as ‘other setting’ (*n* = 1). Details of the screening process are shown in the extended PRISMA 2020 flowchart in (Fig. 2; [17]).

### General characteristics

A total of 11 EEs were identified, of which four studies focused on DCM and five on HCM [24–34]. Two studies included both types of CMs, of which one mainly focussed on DCM patients [32, 34]. Four studies were EEs of diagnostic interventions, while seven studies investigated different treatment options. Identified diagnostic interventions were cascade genetic testing (*n* = 3) and a comparison of two follow-up care programmes for relatives (*n* = 1). Identified treatments were ICDs (*n* = 3), ventricular assist devices (VADs) (*n* = 2), wearable cardioverter defibrillators (WCDs) (*n* = 1) and automated external defibrillators (AEDs) (*n* = 1).

Most EEs were conducted in the USA (*n* = 5), followed by Australia (*n* = 2) and the UK, Sweden, the Netherlands and Japan (*n* = 1, respectively). The majority used the societal perspective for analysis (*n* = 3). Other perspectives were a healthcare system perspective (*n* = 2), and a payer (*n* = 2) or a provider perspective (*n* = 2). Two studies analysed cost-effectiveness from both the societal and healthcare system perspective [24, 31]. The most frequently used study design was a model-based analysis (*n* = 9); the exceptions were two trial-based analyses [32, 34]. One trial-based analysis additionally included a model simulation to account for a longer time period than investigated in their trial [32]. As the primary outcome, QALYs were used in eight studies [24–29, 31, 32]. Two studies chose different outcomes, such as life years saved/gained (LYS/LYG) (*n* = 1) or perceived personal control and patient satisfaction (*n* = 1) [33, 34]. One study reported both QALYs and LYG [30].

In total, two COI studies were identified and included in this review [35, 36]. Both studies focused on HCM and reported hospitalisation-related costs within the US healthcare system setting. One study focussed on obstructive HCM, while the other study focused on HCM patients with and without arrhythmias. For patients with DCM no COI study was found. An overview of the study characteristics is presented in Tab. 1.

**Table 1:** Overview and quality of included studies

Study	Year	Category	CM	Country	Perspective	Interventions	Outcome (2020 Euros)	Conclusion	Quality
*Economic evaluations—Diagnostics*									*88.7%*
Wordsworth [33]	2010	Genetic testing	HCM	UK	Provider	Cascade genetic testing vs clinical surveillance	€ 16,626/LYS	Cascade genetic testing is cost-effective	91.7%
Ingles [30]	2012	Genetic testing	HCM	Australia	Payer	Genetic testing vs clinical screening	€ 484/QALY € 7840/LYG	Cascade genetic testingis cost-effective	92.1%
Catchpool [25]	2019	Genetic testing	DCM	Australia	HCS	Cascade genetic testing vs clinical surveillance	€ 3492/QALY	Cascade genetic testing is cost-effective	94.7%
Nieuwhof [34]	2017	Follow-up care programmes for relatives	HCM and DCM	Netherlands	Provider	Follow-up care by genetic counsellor vs follow-up care by cardiologist	∆ costs of-€ 9.46; ∆ perceived personal control score of 0.11 ∆ proportion of follow-up care provided of 35.7% ∆ patient satisfaction score of 42	Follow-up care by genetic counsellor is much appreciated at lower costs	76.5%
*Economic evaluations—Treatments*									*85.4%*
Evers [26]	2019	WCD	DCM	USA	HCS	[1] Home-no WCD [2] Home-WCD [3] Inpatient stay	[1 vs 2] € 16,543/QALY [3 vs 2] € 210,380/QALY	Use of WCDs is cost-effective	71.1%
Haag [28]	2020	AED	HCM	USA	Societal	At-home AED vs none	€ 69,905/QALY	Use of AEDs is cost-effective	84.2%
Feingold [27]	2010	ICD	DCM	USA	Societal	With and without ICD	€ 284,027/QALY	ICD placement is not cost-effective	84.2%
Haag [29]	2020	ICD	HCM	USA	Societal	With and without ICD	€ 2,433/QALY intermediate risk -€ 6393/QALY high risk	ICD placement is cost-effective	84.2%
Magnusson [31]	2020	ICD	HCM	Sweden	[1] HCS [2] Societal	With and without ICD	[1] € 15,610/QALY [2] -€ 55,405/QALY	ICD placement is cost-effective	92.1%
Takura [32]	2015	VAD	HCM and DCM	Japan	Payer	Implantable VADs vs extracorporeal devices	€ 280,606/QALY (12 months) € 95,088/QALY (36 months)	VADs are cost-effective only in the long term	90.0%
Avanceña [24]	2021	VAD	DCM	USA	[1] Societal [2] HCS	VAD vs watchful waiting	[1] € 159,398/QALY [2] € 136,422/QALY	VADs are not cost-effective	92.1%
*Cost-of-illness studies*									*66.7%*
Jan [35]	2016	–	HCM	USA	HCS	Top-down approach	Mean hospital costs of € 22,703.69 Median € 17,337.75	Highest burden in children and adolescents	50.0%
Tripathi [36]	2018	–	HCM	USA	Provider	Top-down approach	Mean hospital costs (€ 18,998.77 vs € 14,475.43)	Arrhythmias linked to higher economic burden	83.3%

*CM* cardiomyopathy, *HCM* hypertrophic cardiomyopathy, *DCM* dilated cardiomyopathy, *AED* automated external defibrillator, *WCD* wearable cardioverter defibrillator, *ICD* implantable cardioverter defibrillator, *VAD* ventricular assist devices, *HCS* healthcare system, *LYS* life years saved, *LYG* life years gained, *QALY* quality adjusted life years, ∆ incremental.

### Cost-effectiveness plane

**Fig. 3:**
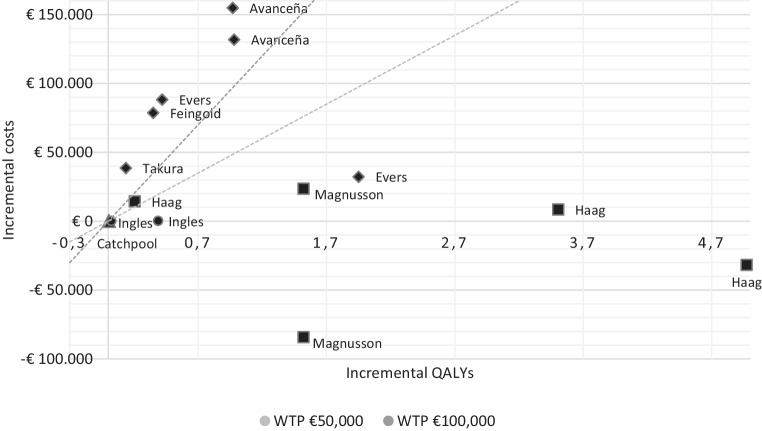
Cost-effectiveness plane of different interventions for hypertrophic and dilated cardiomyopathy. Cost-effectiveness plane with willingness to pay (*WTP*) thresholds of € 50,000 and € 100,000 per quality adjusted life-year (*QALY*) gained: ● Diagnostics in hypertrophic cardiomyopathy in QALYs; ■ Treatments for hypertrophic cardiomyopathy in QALYs; ▲ Diagnostics in dilated cardiomyopathy in QALYs; ♦ Treatments for dilated cardiomyopathy in QALYs

Figure 3 shows the cost-effectiveness plane, including all reported ICERs using QALYs as outcome. Most studies chose a WTP threshold of either € 100,000 or € 50,000 per additional QALY gained [24–30]. The results of most interventions lie in the upper quadrant, indicating more effects but also higher costs relative to the comparator. Diagnostic interventions are located either below or in-between the two thresholds. Treatments for DCM are mostly located above the upper threshold of € 100,000 and are thus not cost-effective. For HCM, treatments are consistently below the € 50,000 threshold, with the exception of one finding. Notably, two results reported by Magnusson and Wimo [31] and Haag et al. [29] are in the lower quadrant, indicating more effects and lower costs relative to the comparator. In this case, the ICER is negative and the intervention is unequivocally cost-effective, making the intervention dominant, and thus more optimal, in relation to the comparator [7].

### Hypertrophic cardiomyopathy

#### Paediatric population

Two model-based EEs, both conducted by Haag et al., are available for children with HCM at intermediate and/or high risk of SCD. In these studies, the cost-effectiveness of (1) at-home AEDs compared to no at-home AEDs in children not eligible for ICD placement and (2) ICDs compared to no ICDs was assessed. From a US societal perspective, Haag et al. conclude that AEDs as well as ICDs are cost-effective in children at intermediate risk of SCD (€ 69,905/QALY and € 2433/QALY, respectively) and emphasise that ICD placements are even the dominant strategy in children at high risk of SCD (-€ 6393/QALY) [28, 29].

#### Adult population

Three model-based EEs are available for an adult population with HCM. Two studies, by Wordsworth et al. [33] and Ingles et al. [30], assessed the cost-effectiveness of cascade genetic testing of relatives compared to periodical clinical surveillance, from a UK and Australian perspective, respectively. Both report results far below the selected WTP thresholds, indicating that genetic testing is likely to be very cost-effective in HCM [30, 33]. Further, Magnusson and Wimo analysed the cost-effectiveness of ICDs in comparison with no ICDs in adults with HCM and a high risk of SCD within a Swedish setting and showed that an ICD placement in this patient population is considered cost-effective from both a healthcare system (€ 15,610/QALY) and a societal perspective (-€ 55,405/QALY). From the latter perspective, ICDs can even be regarded as the dominant strategy [31].

### Dilated cardiomyopathy

#### Paediatric population

Three model-based EEs are available for a paediatric DCM population. All studies were performed from a US healthcare and/or societal perspective, but targeted different interventions. Evers et al. focused on children at high risk of SCD waiting for an ICD placement, and compared the use of home-WCD to (1) no home-WCD and (2) to inpatient stays, with the result that the use of WCDs could be regarded as cost-effective [26]. Feingold et al. analysed the cost-effectiveness of ICDs in children with DCM and symptomatic HF and concluded that ICDs are not cost-effective for these patients, given a threshold of $100,000/QALY [27]. Avanceña et al. looked at VADs in children with stable, inotrope-dependent HF and compared the surgical addition of VADs to watchful waiting, indicating that VADs are neither cost-effective from a healthcare nor from a societal perspective at a WTP of $100,000/QALY [24].

#### Adult population

In an adult DCM patient population, one model-based and two trial-based EEs are available, whereby both trial-based EEs included HCM in addition to DCM patients [32, 34]. Catchpool et al. performed a modelling study and concluded that genetic testing is highly cost-effective from an Australian healthcare system perspective (€ 3492/QALY), suggesting its adoption into routine clinical management for DCM [25]. Nieuwhof et al. focussed on first-degree relatives, mainly those of DCM but also of HCM patients, and performed a randomised evaluation study to compare conventional follow-up care by a cardiologist to that provided by a genetic counsellor (a nurse in the Dutch healthcare setting specifically trained in cardiogenetics). The latter option was thereby considered a feasible care modality, as it achieved higher patient satisfaction at lower costs [34]. Lastly, Takura et al. performed a trial-based analysis and compared extracorporeal devices to surgically implantable VADs in Japanese adults. To account for long-term costs and benefits, an economic model was built in addition. As a result, considering a longer time horizon, VADs might be used as a cost-effective (€ 95,088/QALY) bridge strategy until heart transplantation or as a treatment option if a patient is not eligible for a heart transplant [32].

### COI studies

Two COI studies on HCM were identified and included, of which one focuses particularly on obstructive HCM [35, 36]. For patients with obstructive HCM, the average length of hospital stay was 4.9 days [35]. Overall, the median costs for a hospital admission with obstructive HCM were € 17,337.75 [35]. The highest average hospital costs, of € 29,663.96 per admission, were reported for the paediatric patient population [35]. Tripathi et al. reported that the presence of arrhythmias generally leads to higher hospitalisation costs [36]. HCM patients with arrhythmias incurred total average hospital costs of € 18,998.77, whereas for HCM patients without arrhythmias significantly lower mean hospital costs of € 14,475.43 were reported [36]. The highest hospital costs arise in patients suffering from ventricular fibrillation, namely € 36,205.24, and ventricular tachycardia, with a total of € 26,843.80 [36]. Types of treatment or standard deviations were not provided in any study. Referring to the current literature, the average length of hospital stay for obstructive HCM patients undergoing septal ablation or myectomy was 3.4 days and 8.9 days, respectively, with average hospitalisation costs of around € 17,730.23 and € 39,425.18 [37, 38]. According to Tripathi et al., the cost of care for HCM patients increased significantly over time, although the average length of stay remained equal over the study period. The increasing prevalence of arrhythmia and the use of more expensive treatments were assumed to be underlying causes [36].

### Quality assessment

The CHEC list critically assessed included studies regarding their study characteristics, methodology and reporting [6, 16, 23]. The total mean quality score of all EEs was 86.6%, ranging from 71.1% to 94.7%. Only two studies scored below 80%, indicating that the overall quality of EEs is high [26, 34]. EEs of diagnostic interventions reached a higher mean quality in comparison with EEs of treatments (88.7% vs 85.4%, respectively). In particular, studies focusing on cascade genetic testing achieved a high mean quality of 92.8% [25, 30, 33]. Two of these studies were performed in Australia and one in the UK. Regarding COI studies, the mean quality score was 66.7%. Details concerning the quality assessment can be found in Section B of the Electronic Supplementary Material.

## Discussion

This systematic literature review provides an overview of current knowledge about the economic and societal burden of HCM and DCM.

A huge knowledge gap was identified regarding COI studies, as only two US studies on HCM were found, and both of these suffer from methodological issues. Jan and colleagues reported higher average hospital-related costs than Tripathi et al., but did not account for possible comorbidities which, according to Larg and Moss [6], might impact average costs. Both refer to cross-sectional hospital charges while leaving out costs for other medical care, costs for patients and caregivers, and productivity losses. Furthermore, the specific types of interventions performed were not reported. This is in contrast to broader populations, such as HF patients, where various COI studies with a good methodological quality have been identified [8]. According to Urbich et al. [8], hospital-related costs for HF were identified as cost drivers, with median costs of € 12,838.78 per patient, which is slightly lower than the amounts reported by Jan et al. and Tripathi et al.

Few EEs of HCM and DCM are available in the current literature; these show that the cost-effectiveness varies between interventions and patient subgroups. The overall study quality was assessed as high, with only two studies yielding a quality score below 80% [26, 34]. This is in line with the quality reported in the systematic reviews of Colquitt et al. and Gialama et al. on the cost-effectiveness of ICDs [9, 10]. Regarding inherited CMs, most studies reported a lack of data, making the studies heavily reliant on assumptions and expert opinions [24–29]. To account for uncertainty, intensive sensitivity analyses were performed in 9 of 11 included studies. Most studies decreased in quality due to neglecting relevant costs. Productivity losses and the burden for caregivers were missing throughout most studies, mainly due to the selection of too narrow perspectives. In view of the relatively young age of the patients, productivity losses in particular might impact the analysis, since they can account for a huge amount of additional costs over a lifetime [24, 31]. Furthermore, not all studies considered the impact on the quality of life of patients and families. While most studies used QALYs as the outcome, Wordsworth et al. used LYS which, as described by Drummond et al. [7], are incomplete measures since they account only for the extension of life through certain interventions, but not for the quality of life during life extension.

Due to large differences between study characteristics in terms of perspectives, assumptions and outcomes, a direct comparison between studies is difficult. Further, costs and outcomes might vary considerably across diseases (HCM or DCM) and patient subgroups (age and symptomatology), requiring studies to be assessed separately. The cost-effectiveness of interventions hence strongly depends on the selected patient subgroup; however, only a few studies were identified per subgroup. For interventions like genetic testing or external defibrillator devices there is a consensus about cost-effectiveness despite there being different targeted subgroups. With regard to treatments like ICDs or VADs, there is mixed consensus or there are notable differences between study populations. Colquitt et al. and Gialama et al. concluded that ICDs might be cost-effective in HF patients at high risk of SCD, but found mixed evidence in populations at lower risk of SCD [9, 10]. For VADs, studies report similar findings, suggesting that VADs are cost-effective in adults but less so in children with advanced HF, supporting evidence found by Avanceña et al. and Takura et al. [24, 32, 39, 40]. Generally, the findings of this review are in line with results reported for HF patients. However, HF patients can have different underlying heart conditions, which questions the generalisability of these results [9]. Due to different disease characteristics, the application of the study results to other inheritable CMs, such as arrhythmogenic right ventricular CMs, is limited [1]. Furthermore, the transferability of results to middle- or low-income countries remains difficult due to considerable differences in healthcare systems [7].

### Strengths and limitations

Several strengths of this review need to be highlighted. First, validated research filters were selected to ensure a complete identification process [21]. Second, quality scores were calculated to critically appraise the informative value of the included studies. For transparency, detailed results of the quality assessment were presented on the study and item level. Third, double scoring was performed by at least two independent reviewers to ensure correct study selection and assessment procedures. Fourth, the publication of the review in PROSPERO and adherence to the PRISMA guidelines guaranteed full transparency and open access to relevant study details.

The present review also has some limitations. First, interventions for inherited and acquired CMs might be similar, making clear differentiation difficult [10]. This issue was addressed by keeping the search strategy broad and by selecting more sensitive rather than precise search filters. Second, an exact distinction between familial and non-familial CMs was not always possible, due to missing information about the study population or to methodological limitations of the economic models [24, 26–29, 31, 32]. To account for this issue, clear eligibility criteria were formulated and strictly followed during the entire selection process. Thereby, only studies referring to HCM, DCM or relatives at risk were included. Studies on broader patient populations, e.g. athletes, or on the general population, which were often used in EEs of different screening strategies in HCM, were regarded as irrelevant [41–45]. Third, the CHEC list uses similar weights for every item. However, we acknowledge that some items could be more important than others, potentially affecting the overall quality of the studies. In the absence of guidelines for different weights, we retained equal weighting.

## Conclusion

COI studies and EEs provide important information needed for making decisions in healthcare [6, 7]. To sufficiently inform and support policy-makers in decisions about the clinical management of HCM and DCM, more research is necessary. In particular, only two COI studies were identified; further studies are needed to gain more insights into the economic consequences and key cost drivers in HCM and DCM. Although cost-effectiveness has been examined in a few EEs, not all relevant cost types were considered in all studies. Due to patient heterogeneity, the cost-effectiveness of interventions is strongly reliant on the targeted patient populations. As evidence is limited, more studies in different settings are needed to confirm the results of available EEs. Future studies need more methodological standardisation to enhance comparability between studies. More concretely, studies should consider the quality of life of patients by using QALYs. Moreover, a broader perspective on costs is needed to include the full economic impact of DCM and HCM, and to draw valid conclusions about the optimal care for DCM and HCM patients.

## Supplementary Information

Section A of the Electronic Supplementary Material entails details on the search strategy (Tab. 1–5). Section B includes the quality assessment checklists (Tab. 1–2) and the results of the study quality assessment on item level (Tab. 3).
