# Supplementary material for: A systematic literature review of economic evaluations and cost-of-illness studies of inherited cardiomyopathies
Source: Neth Heart J. 2023 May 12;31(6):226–37. doi: 10.1007/s12471-023-01776-1 (PMC10188671; doi:10.1007/s12471-023-01776-1)
Supplement: Supplementary file 1 — Section A of the Electronic Supplementary Material entails details on the search strategy (Tab. 1–5). Section B includes the quality assessment checklists (Tab. 1–2) and the results of the study quality assessment on item level (Tab. 3). [file 12471_2023_1776_MOESM1_ESM.docx]

# Supplementary material – A) Search strategy

**Table 1:** MEDLINE (Ovid)

| # | Keywords | Results |
| --- | --- | --- |
| 1 | Cardiomyopath*.ti,ab,kf. | 76,497 |
| 2 | Economics/ or exp "Costs and Cost Analysis"/ or Economics, Nursing/ or Economics, Medical/ or Economics, Pharmaceutical/ or exp Economics, Hospital/ or Economics, Dental/ or exp "Fees and Charges"/ or exp Budgets/ or budget*.ti,ab,kf. or (economic* or cost or costs or costly or costing or price or prices or pricing or pharmacoeconomic* or pharmaco-economic* or expenditure or expenditures or expense or expenses or financial or finance or finances or financed).ti,kf. or (economic* or cost or costs or costly or costing or price or prices or pricing or pharmacoeconomic* or pharmaco-economic* or expenditure or expenditures or expense or expenses or financial or finance or finances or financed).ab. /freq=2 or (cost* adj2 (effective* or utilit* or benefit* or minimi* or analy* or outcome or outcomes)).ab,kf. or (value adj2 (money or monetary)).ti,ab,kf. or exp models, economic/ or economic model*.ab,kf. or markov chains/ or markov.ti,ab,kf. or monte carlo method/ or monte carlo.ti,ab,kf. or exp Decision Theory/ or (decision* adj2 (tree* or analy* or model*)).ti,ab,kf. | 771,785 |
| 3 | ((cost adj2 illness) or (cost* adj2 disease) or ("spending*" or "healthcare use" or "healthcare usage" or "health care use" or "health care usage" or "healthcare resource utili?ation" or "health care resource utili?ation" or "health care resource use" or "healthcare resource use" or "resource utili?ation" or "productivity loss*" or "disease burden" or "economic burden" or "societal burden" or "socio-economic burden")).ti,ab,kf. | 66,473 |
| 4 | 2 or 3 | 807,040 |
| 5 | 1 and 4 | 701 |
| 6 | limit 5 to (humans and yr="2010 -Current") | 358 |

**Date of insertion:** 28.04.2021

**Search filter:** Sensitive economic evaluation search filter for MEDLINE (Ovid)
- CADTH database search filters. Ottawa: CADTH; 2016.

https://www.cadth.ca/resources/finding-evidence/strings-attached-cadths-database-search-filters#eco

**Table 2:** EMBASE (Ovid)

| # | Keywords | Results |
| --- | --- | --- |
| 1 | Cardiomyopath*.ti,ab,kw. | 124,812 |
| 2 | Economics/ or Cost/ or exp Health Economics/ or (economic* or cost or costs or costly or costing or price or prices or pricing or pharmacoeconomic* or pharmaco-economic* or expenditure or expenditures or expense or expenses or financial or finance or finances or financed).ti,kw. or (economic* or cost or costs or costly or costing or price or prices or pricing or pharmacoeconomic* or pharmaco-economic* or expenditure or expenditures or expense or expenses or financial or finance or finances or financed).ab. /freq=2 or (cost* adj2 (effective* or utilit* or analy* or outcome or outcomes)).ab,kw. or (value adj2 (money or monetary)).ti,ab,kw. or Statistical Model/ or economic model*.ab,kw. or Probability/ or markov.ti,ab,kw. or monte carlo method/ or monte carlo.ti,ab,kw. or Decision Theory/ or Decision Tree/ or (decision* adj2 (tree* or analy* or model*)).ti,ab,kw. | 1,705,735 |
| 3 | ((cost adj2 illness) or (cost* adj2 disease) or ("spending*" or "healthcare use" or "healthcare usage" or "health care use" or "health care usage" or "healthcare resource utili?ation" or "health care resource utili?ation" or "health care resource use" or "healthcare resource use" or "resource utili?ation" or "productivity loss*" or "disease burden" or "economic burden" or "societal burden" or "socio-economic burden")).ti,ab,kw. | 103,902 |
| 4 | 2 or 3 | 1,755,900 |
| 5 | 1 and 4 | 2,412 |
| 6 | limit 5 to (human and yr="2010 -Current") | 1,734 |

**Date of insertion:** 28.04.2021

**Search filter:** Sensitive economic evaluation search filter for EMBASE (Ovid) - Canadian Agency for Drugs and Technologies in Health (CADTH) sensitive filter

https://www.cadth.ca/resources/finding-evidence/strings-attached-cadths-database-search-filters#eco

**Table 3:** NHS EED

| # | Keywords | Results |
| --- | --- | --- |
| MeSh Search 1 | MeSH DESCRIPTOR Cardiomyopathy, Dilated EXPLODE 1 2 3 IN NHSEED | 8 |
| MeSH Search 2 | MeSH DESCRIPTOR Cardiomyopathy, Hypertrophic EXPLODE 2 1 IN NHSEED | 3 |
| MeSH Search 3 | MeSH DESCRIPTOR Cardiomyopathy, Hypertrophic, Familial EXPLODE 3 2 1 IN NHSEED | 2 |
| MeSH Search 4 | MeSH DESCRIPTOR Cardiomyopathies EXPLODE 1 IN NHSEED | 2 |
| Sum | | 15 |
| Filter | From 2010-2021 | 10 excluded |
| End sum | | 5 |

**Date of insertion:** 26.04.2021

**Search filter:** None

**Table 4:** EconLit (EBSCO)

| # | Keywords | Option | Results |
| --- | --- | --- | --- |
| 1 | Cardiovascular or cardiomyopath* | All text |  |
| AND | | | |
| 2 | Cost-effectiveness or economic model or economic impact or cost utility or economic evaluation or burden | All text |  |
| 3 | From 2010 - 2021 |  | 59 |

**Date of insertion:** 28.04.2021

**Search filter:** None

**Table 5:** Web of Science (Core collection)

| # | Keywords | Results |
| --- | --- | --- |
| 1 | TS= (cardiomyopath*) | 108,102 |
| 2 | TS= (economic$ OR “costs and cost analysis” OR pric* OR financ* OR “economic model*” OR “cost-utilit*” OR “health utilit*” OR “economic evaluation*” OR “economic review*” OR “cost outcome$” OR "cost minimi?ation" OR “cost analys*” OR “economic analys*” OR “cost-effective*” OR "cost-benefit" OR budget OR "budget impact" OR “monetary value” OR “life year” OR “life years” OR qaly* OR pharmacoeconomic* OR “pharmaco-economic*” OR “cost-effective*” OR pharmacoeconomic* OR “pharmaco-economic*” OR “cost-effectiveness analys*” OR “markov” OR "monte carlo" or "decision tree" OR "decision model" OR “decision analys?s” OR “statistical model”) | 2,376,256 |
| 3 | TS= ("cost of illness" OR "cost of disease" OR "healthcare use" OR "healthcare usage" OR expenditure$ OR expense$ OR "resource utili?ation" OR "burden of disease" OR "economic burden" OR "societal burden" OR "disease burden" OR spending$ OR "health* spending*" OR "healthcare cost$" OR "health care cost$" OR "health resource utili?ation" OR "healthcare resource utili?ation" OR "health resource use" OR "healthcare resource use" OR "healthcare resource usage" OR "health resource usage" OR "resource cost$" OR "productivity loss*" OR "cost trend") | 451,121 |
| 4 | #2 OR #3 | 2,715,909 |
| 5 | #1 AND #4 | 1,278 |
| 6 | #5  Timespan: 2010 - 2021 | 875 |

**Date of insertion:** 28.04.2021

**Search filter:** Not available for Web of Science; sensitive CADTH filter for economic evaluations was adapted as much as possible.

# Supplementary material – B) Quality assessment

**Table 1:** Extended CHEC-list for the quality assessment of economic evaluations
(Evers et al., 2005; Wijnen et al. 2016)

|  | CHEC-extended | Yes | No |
| --- | --- | --- | --- |
| 1. | Is the study population clearly described? |  |  |
| 2. | Are competing alternatives clearly described? |  |  |
| 3. | Is a well-defined research question posed in answerable form? |  |  |
| 4. | Is the economic study design appropriate to the stated objective? |  |  |
| 5. | Are the structural assumptions and the validation methods of the model properly reported? |  |  |
| 6. | Is the chosen time horizon appropriate in order to include relevant costs and consequences? |  |  |
| 7. | Is the actual perspective chosen appropriate? |  |  |
| 8. | Are all important and relevant costs for each alternative identified? |  |  |
| 9. | Are all costs measured appropriately in physical units? |  |  |
| 10. | Are costs valued appropriately? |  |  |
| 11. | Are all important and relevant outcomes for each alternative identified? |  |  |
| 12. | Are all outcomes measured appropriately? |  |  |
| 13. | Are outcomes valued appropriately? |  |  |
| 14. | Is an appropriate incremental analysis of costs and outcomes of alternatives performed? |  |  |
| 15. | Are all future costs and outcomes discounted appropriately? |  |  |
| 16. | Are all important variables, whose values are uncertain, appropriately subjected to sensitivity analysis? |  |  |
| 17. | Do the conclusions follow from the data reported? |  |  |
| 18. | Does the study discuss the generalizability of the results to other settings and patient/client groups? |  |  |
| 19. | Does the article/report indicate that there is no potential conflict of interest of study researcher(s) and funder(s)? (transparency) |  |  |
| 20. | Are ethical and distributional issues discussed appropriately? |  |  |

* Q4: Always yes otherwise exclude

* Q13: Only applicable for CBA and CUA

**Table 2:** Modified CHEC-list for the quality assessment of COI
(Evers et al., 2005; Larg & Moss, 2011)

|  | Preliminary checklist for the quality assessment of cost-of-illness (COI) studies: | Yes | No |
| --- | --- | --- | --- |
| Study characteristics | | | |
| 1. | Is the study population clearly described? |  |  |
| 2. | Is a well-defined research question posed in answerable form? |  |  |
| 3. | Is the actual perspective chosen appropriate to the stated objective? |  |  |
| Methodology and cost analysis | | | |
| 4. | Is the chosen time horizon specified and appropriate to include all relevant costs? |  |  |
| 5. | Are all important and relevant costs identified? |  |  |
| 6. | Are all costs measured appropriately? |  |  |
| 7. | Are costs valued appropriately? |  |  |
| 8. | Are all future costs discounted appropriately? |  |  |
| 9. | Are all important variables, whose values are uncertain, subjected to sensitivity analysis? |  |  |
| Results and reporting | | | |
| 10. | Do the conclusions follow from the data reported? |  |  |
| 11. | Does the study discuss the generalizability, comparing the results to patient/client groups in other settings? |  |  |
| 12. | Does the article indicate that there is (no) potential conflict of interest? |  |  |
| 13. | Does the study discuss important limitations regarding the cost components, data, assumptions, and methods? |  |  |

**Sources:**

Criteria 1-12:

Evers, S.; Goossens, M.; Vet, H. de; van Tulder, M.; Ament, A. (2005): Criteria list for assessment of methodological quality of economic evaluations. Consensus on Health Economic Criteria. In: International journal of technology assessment in health care 21 (2), S. 240–245. DOI: 10.1017/S0266462305050324.

Criteria 13:

Larg, A.; Moss, J. R. (2011): Cost-of-illness studies. A guide to critical evaluation. In: *PharmacoEconomics* 29 (8), S. 653–671. DOI: 10.2165/11588380-000000000-00000.

**Table 3:** Quality assessment of eligible studies

| Quality assessment of economic evaluations, using the extended CHEC list | | | | | | | | | | | | | | | | | | | | | Score |
| --- | --- | --- | --- | --- | --- | --- | --- | --- | --- | --- | --- | --- | --- | --- | --- | --- | --- | --- | --- | --- | --- |
| Item | Q1 | Q2 | Q3 | Q4 | Q5 | Q6 | Q7 | Q8 | Q9 | Q10 | Q11 | Q12 | Q13 | Q14 | Q15 | Q16 | Q17 | Q18 | Q19 | Q20 | **%** |
| Avanceña | Yes | Yes | Sub | Yes | Yes | Yes | Yes | Yes | Sub | Yes | Yes | Yes | Yes | Yes | Yes | Yes | Yes | Yes | Sub | Na | 92.1 |
| Catchpool | Yes | Yes | Yes | Yes | Yes | Yes | Sub | Yes | Yes | Yes | Yes | Yes | Yes | Yes | Yes | Yes | Yes | Yes | Sub | Na | 94.7 |
| Evers | Sub | Yes | Yes | Yes | Sub | Yes | Sub | Yes | No | Sub | Yes | Yes | Yes | Yes | Sub | Yes | Yes | No | No | Na | 71.1 |
| Feingold | Yes | Yes | Yes | Yes | Yes | Sub | Yes | No | Sub | Sub | Yes | Sub | Yes | Yes | Yes | Yes | Yes | Yes | Yes | Na | 84.2 |
| Haag (AED) | Yes | Yes | Yes | Yes | Yes | Yes | Yes | Sub | Sub | Sub | Yes | Sub | Yes | Yes | Sub | Yes | Yes | Yes | Sub | Na | 84.2 |
| Haag (ICD) | Yes | Yes | Yes | Yes | Sub | Yes | Yes | Sub | Sub | Sub | Yes | Sub | Yes | Yes | Sub | Yes | Yes | Yes | Yes | Na | 84.2 |
| Ingles | Yes | Yes | Yes | Yes | Yes | Yes | No | Yes | Yes | Yes | Yes | Yes | Yes | Yes | Yes | Yes | Yes | Sub | Yes | Na | 92.1 |
| Magnusson | Yes | Yes | Yes | Yes | Yes | Sub | Yes | Yes | Sub | Yes | Yes | Yes | Yes | Yes | Yes | Sub | Yes | Yes | Yes | Na | 92.1 |
| Nieuwhof | Yes | Yes | Yes | Yes | Na | Yes | No | Sub | Yes | Sub | Sub | Yes | Na | No | Na | Yes | Yes | Yes | Sub | Yes | 76.5 |
| Takura | Yes | Yes | Yes | Yes | Sub | Yes | Sub | Yes | Yes | Yes | Yes | Yes | Yes | Yes | Yes | No | Yes | Yes | Yes | Yes | 90.0 |
| Wordsworth | Yes | Yes | Yes | Yes | Yes | Yes | No | Yes | Yes | Yes | Sub | Yes | Na | Yes | Yes | Yes | Yes | Yes | Yes | Na | 91.7 |
| Overall mean quality score | | | |  |  |  |  |  |  |  |  |  |  |  |  |  |  |  |  |  | **86.6** |
|  | | | | | | | | | | | | | | | | | | | | |  |
| Quality assessment of cost-of-illness studies, using a modified CHEC list | | | | | | | | | | | | | | | | | | | | | **Score** |
| Item | Q1 | Q2 | Q3 | Q4 | Q5 | Q6 | Q7 | Q8 | Q9 | Q10 | Q11 | Q12 | Q13 |  | | | | | | | % |
| Jan | Yes | Yes | No | Yes | No | No | Yes | Na | No | Yes | No | Yes | No |  |  |  |  |  |  |  | 50.0 |
| Tripathi | Yes | Yes | Yes | Yes | Yes | No | Yes | Na | No | Yes | Yes | Yes | Yes |  |  |  |  |  |  |  | 83.3 |
| Overall mean quality score | | | |  |  |  |  |  |  |  |  |  |  |  |  |  |  |  |  |  | **66.7** |

**Abbreviations:** CHEC = Consensus on Health Economic Criteria, Q = Question, Na = Not applicable, Sub = suboptimal answered, AED = automated external defibrillator, ICD = Implantable cardioverter defibrillator
